# A dietary anthocyanin cyanidin-3-*O*-glucoside binds to PPARs to regulate glucose metabolism and insulin sensitivity in mice

**DOI:** 10.1038/s42003-020-01231-6

**Published:** 2020-09-18

**Authors:** Yaoyao Jia, Chunyan Wu, Young-Suk Kim, Seung Ok Yang, Yeonji Kim, Ji-Sun Kim, Mi-Young Jeong, Ji Hae Lee, Bobae Kim, Soyoung Lee, Hyun-Seok Oh, Jia Kim, Min-Young So, Ye Eun Yoon, Trung Thanh Thach, Tai Hyun Park, Sung-Joon Lee

**Affiliations:** 1 Department of Biotechnology, School of Life Sciences and Biotechnology, BK21 PLUS. Korea University, Seoul, 02841 Republic of Korea; 2 Department of Food Science and Engineering, Ewha Womans University, Seoul, 03760 Republic of Korea; 3 School of Chemical and Biological Engineering, Seoul National University, Seoul, 08826 Republic of Korea

**Keywords:** Agriculture, Metabolic syndrome, Fats, Biomedical materials, Metabolic syndrome

## Abstract

We demonstrate the mechanism by which C3G, a major dietary anthocyanin, regulates energy metabolism and insulin sensitivity. Oral administration of C3G reduced hepatic and plasma triglyceride levels, adiposity, and improved glucose tolerance in mice fed high-fat diet. Hepatic metabolomic analysis revealed that C3G shifted metabolite profiles towards fatty acid oxidation and ketogenesis. C3G increased glucose uptake in HepG2 cells and C2C12 myotubes and induced the rate of hepatic fatty acid oxidation. C3G directly interacted with and activated PPARs, with the highest affinity for PPARα. The ability of C3G to reduce plasma and hepatic triglycerides, glucose tolerance, and adiposity and to induce oxygen consumption and energy expenditure was abrogated in PPARα-deficient mice, suggesting that PPARα is the major target for C3G. These findings demonstrate that the dietary anthocyanin C3G activates PPARs, a master regulators of energy metabolism. C3G is an agonistic ligand of PPARs and stimulates fuel preference to fat.

## Abstract (web summary)

Jia, Wu, Kim et al. show that cyanidin-3-O-glucoside (C3G), a major dietary anthocyanin, functions as a ligand for PPARalpha to regulate energy metabolism and insulin sensitivity in mouse models of obesity and diabetes. This study suggests that C3G slows down the metabolism of glucose, making it a promising therapeutic agent.

## Introduction

Cyanidin-3-*O*-glucoside (C3G) is a major flavonoid anthocyanin in plant-based foods, such as leafy vegetables, berries, red cabbages, teas, and coloured grains^1,2^. Epidemiologic studies have suggested that the dietary intake of anthocyanidins prevents dyslipidaemia, cardiovascular disease, and type 2 diabetes^3–8^. However, the mechanism of action and target proteins of C3G are not clearly understood.

In plants, C3G is an important secondary metabolite with a multitude of biological functions, including UV protection, pathogen defence, insect attraction, symbiosis, and flower variation^9,10^. Plant food containing C3G that is ingested by humans undergoes digestion in the gut, following which both C3G and its aglycone, cyanidin, can be absorbed by the intestinal epithelium and delivered to the circulation to exert multiple biological functions in tissues^11^. C3G is more stable than its aglycone cyanidin in aqueous solution^12^; thus, C3G is believed to be the major bioavailable and active form of cyanidin in human tissues.

Natural small molecules and dietary compounds often exert several biological activities. For example, salicylate, an ancient drug that was originally identified in willow tree bark, carries out multiple biological activities including exerts anti-inflammatory activity^13,14^, reducing hepatic glucose production and lowering adiposity^15^. Short-chain fatty acids interact with at least three G-protein coupled receptors, FFAR2, FFAR3, and the ectopic olfactory receptor OR51E2^16,17^ to have several effects in mice and humans. Likewise, a number of in vitro and in vivo studies reported several biological activities of C3G^18–20^; however, none of these studies have clearly reported the molecular mechanism of action and direct molecular targets of C3G.

Peroxisome-proliferator activated receptors (PPARs) are nuclear receptors and ligand-activated transcription factors that regulates several biological pathways including cellular energy metabolism and inflammation^21^. Three PPARs, -α, -γ, and -δ isoforms have unique tissue distributions and biological activities. PPARα is widely expressed in different tissues with high expression levels in liver and skeletal muscle that regulates the expression of genes encoding enzymes and transport proteins that control lipid homeostasis and thus stimulates fatty acid oxidation and improves lipoprotein metabolism^22,23^. In addition, PPARα has been suggested to play a major role in the regulation of hepatic lipid oxidation and to serve as a distinctive marker of the brown fat phenotype, which is involved in transcriptional control of PGC-1α in brown adipose tissue, thereby maintaining energy homeostasis^24^. PPARα agonists, fibrates, are used to treat hypertriglyceridemia. PPARγ is expressed in several tissues with high expression levels in white adipose tissue and has been known to regulate adipocyte differentiation, fatty acid storage, and glucose metabolism^25^. PPARγ activation improves insulin resistance by opposing the effect of TNFα in adipocytes^26^. PPARγ enhances the expression of a number of genes encoding proteins involved in glucose and lipid metabolism^25^. Thus, PPARγ agonists, thiazolidinediones, are used as prescribed drugs for the treatment of type 2 diabetes.

It has been shown that C3G could increase gene expressions of PPARs^27–29^, therefore, in this study, we investigated whether PPARs, which regulates hepatic lipid metabolism and glucose homeostasis, are molecular targets of C3G. Furthermore, we examined the mechanism of action and the metabolic effects of C3G.

## Results

### C3G reduced dyslipidemia and hyperglycemia in HFD-fed mice

**Fig. 1:**
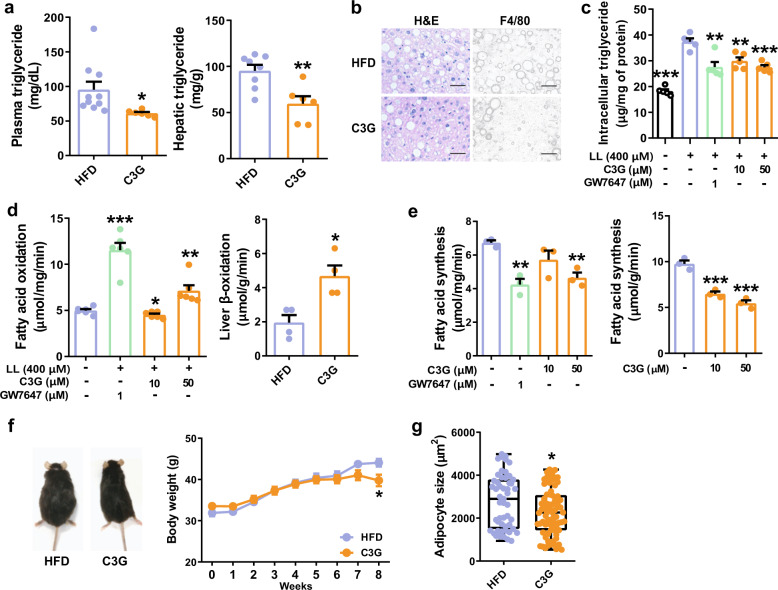
C3G reduces hepatic and plasma triglyceride concentrations and adiposity in mice. Mice were orally administered C3G (50 mg/day/body weight) for 8 weeks and fed a HFD (45% calories from fat). **a** Plasma and hepatic triglyceride concentrations in mice. **b** Representative images of H&E and F4/80 staining of mouse livers in the HFD and C3G groups. Scale Bar: 50 µm **c** Intracellular triglyceride levels. HepG2 cells were stimulated with C3G (10 and 50 μM) and GW7647 (PPARα agonist, 1 μM) for 24 h, and triglyceride levels were measured by an enzymatic method. LL lipid-loading. **d** Measurement of fatty acid oxidation and fatty acid synthesis rates in HepG2 cells treated with C3G or GW7647 and the livers of mice in the HFD and C3G groups. **e** Measurement of the fatty acid synthesis rate in HepG2 cells treated with C3G or GW7647 and in the livers of mice administered C3G. Fatty acid oxidation in the livers was assessed using livers of control and C3G mice while fatty acid synthesis in the livers was measured in liver homogenates treated with vehicle (double distilled water) or two concentrations of C3G. **f** C3G reduces mouse body weight. **g** Box plot of the mean adipocyte size of control and C3G mice. *P < 0.05, **P < 0.01, and ***P < 0.005 compared with controls. HepG2 cells were lipid-loaded prior to the experiment as described in the methods.

First, we performed mouse feeding studies with oral administration of C3G to mice fed a high-fat diet (HFD). The oral administration of C3G for 8 weeks significantly reduced plasma and hepatic triglyceride (TG) concentrations (*P* < 0.05 and *P* < 0.01, respectively); C3G reduced the number of intracellular lipid droplets, and F4/80 levels in C3G-HFD livers, suggesting that C3G improves non-alcoholic fatty liver disease (NAFLD) (Fig. 1a, b). Intracellular TG concentrations were also reduced in HepG2 cells stimulated with C3G compared to control HepG2 cells (Fig. 1c). GW7647, a PPARα agonist, reduced intracellular triglyceride levels and fatty acid oxidation rate but increased fatty acid synthesis rate in lipid-loaded HepG2 cells. Total cholesterol, low-density lipoprotein (LDL)-cholesterol, and high-density lipoprotein (HDL)-cholesterol concentrations were unaltered by C3G administration in mice (Supplementary Fig. 1a). By analysing hepatic fatty acid metabolic rates, C3G was shown to stimulate the rate of fatty acid oxidation but suppress the rate of fatty acid synthesis in both HepG2 cells and mouse livers (Fig. 1d, e). The oral administration of C3G had an antiobesogenic effect on mice, as indicated by reduced body weight, visceral fat tissue weight, adipocyte size, the ratio of white-to-brown adipose tissues and the increased ratio of white adipose tissue-to-skeletal muscle weight in HFD mice administered C3G (Fig. 1f, g, Supplementary Fig. 1b, and Supplementary Table 1). Aortic atherosclerotic plaque formation was decreased in mice administered C3G with a HFD (Supplementary Fig. 1c).

**Fig. 2:**
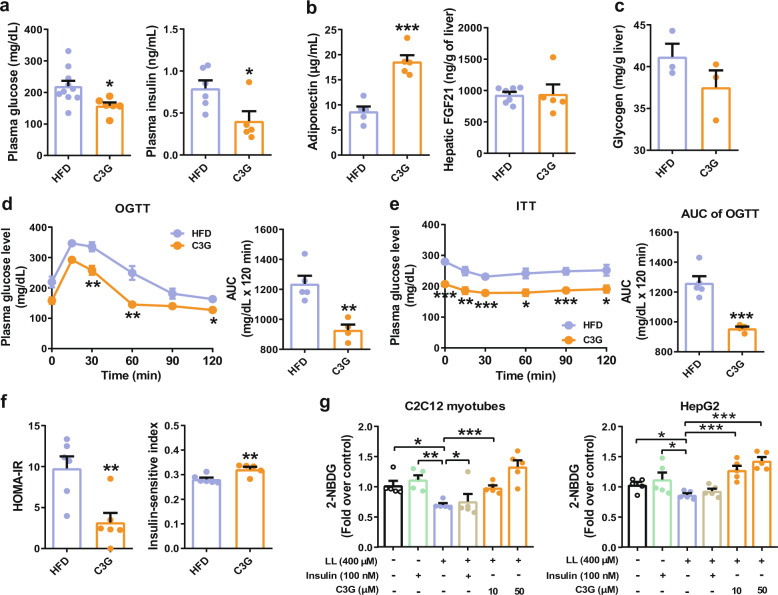
C3G improves glucose and insulin tolerance in mice. **a** Fasting glucose and insulin levels. **b**, **c** Fasting adiponectin, hepatic FGF12, and hepatic glycogen levels in mice. **d** Oral glucose tolerance test. **e** Intraperitoneal insulin tolerance test. **f** Insulin sensitivity indices. **g** Glucose uptake. LL lipid-loading. *P < 0.05, **P < 0.01, and ***P < 0.005 compared with controls.

The oral administration of C3G improved key parameters indicating insulin sensitivity in mice. C3G decreased fasting plasma glucose and insulin concentrations and increased adiponectin concentrations without affecting liver glycogen and FGF21 levels (Fig. 2a–c). C3G also improved glucose and insulin tolerance, HOMA-IR, and the insulin sensitivity index in mice fed a HFD (Fig. 2d–f). To investigate glucose uptake upon treatment with C3G, we induced insulin resistance in cultured C2C12 myotubes and HepG2 cells by treating them with 400 µM lipids for 24 h and then measured their glucose uptake. Insulin promoted glucose uptake in C2C12 myotubes and HepG2 cells that were not lipid-loaded. C3G improved the impaired glucose uptake in lipid-loaded C2C12 myoblasts and HepG2 cells (Fig. 2g). These results collectively demonstrate that C3G improved NAFLD, adiposity, glucose tolerance, hyperglycaemia, and insulin sensitivity in mice fed a HFD.

### C3G alters the hepatic fatty acid oxidation metabolites

**Fig. 3:**
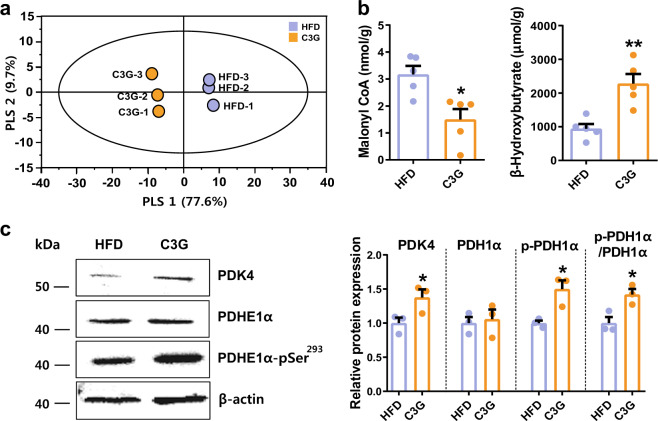
Key metabolite profile in the liver of HFD mice administered C3G. **a** PLSR analysis of liver metabolomes of mice fed a HFD. **b** Levels of malonyl-CoA and β-hydroxybutyrate. **c** Expression of PDK4 and phosphorylation of PDH1α. *P < 0.05 and ***P < 0.01 compared with controls.

We next examined the hepatic metabolomes of fasting mice by CE-MS and GC-TOF-MS and by assay kits. HFD-fed mice were orally administered either vehicle (saline) or C3G for 8 weeks, and the liver metabolome was analysed in mouse livers in a fasting state. CE-MS and GC-TOF-MS analyses identified 92 metabolites (selected key metabolites are shown in Table 1 and Supplementary Table 1). Multivariate statistical analysis was conducted to determine the significance of any differences in the metabolomes of liver tissues from mice fed different diets for 8 weeks. By using partial least squares regression (PLSR) analysis, the metabolite profiles of each group were distinctively clustered. These findings suggest that C3G caused unique and specific alterations in the mouse liver metabolome (Fig. 3a).

**Table 1:** Major liver metabolites from mice fed a HFD and C3G for 8 weeks using CE-MS and GC-TOF-MS.

Metabolites	CE-MS relative peak area (mean ± SD)^a^	
	HFD^b^	C3G^c^
Glycolysis		
Glucose 6-phosphate	25.445 ± 0.567	29.021 ± 0.610*
Fructose 6-phosphate	8.591 ± 0.176	10.051 ± 0.273*
Fructose 1,6-diphosphate	0.653 ± 0.067	0.635 ± 0.100
Dihydroxyacetone phosphate	8.268 ± 0.338	11.394 ± 0.098*
3-Phosphoglyceric acid	70.567 ± 1.293	41.054 ± 0.929*
2-Phosphoglyceric acid	7.956 ± 0.195	4.822 ± 0.134*
Phosphoenolpyruvic acid	17.815 ± 0.337	10.217 ± 0.095*
Pyruvic acid	54.875 ± 1.452	30.684 ± 2.973*
Lactic acid	10140.602 ± 101.363	9024.705 ± 350.355*
Fructose 1-phosphate	1.944 ± 0.776	1.348 ± 0.737*
Glycerol 3-phosphate	230.470 ± 6.285	779.513 ± 25.265*
2,3-Diphosphoglyceric acid	4.747 ± 0.057	0.409 ± 0.024*
TCA cycle		
Acetyl CoA	0.032 ± 0.013	0.027 ± 0.010
CoA	0.412 ± 0.012	0.505 ± 0.028*
Citric acid	3.995 ± 0.455	N.D.
2-Hydroxyglutaric acid	12.884 ± 0.588	8.084 ± 1.471*
Succinic acid	62.239 ± 1.267	135.582 ± 4.424*
Fumaric acid	700.874 ± 9.309	599.059 ± 16.568*
Malic acid	2106.790 ± 28.503	1795.026 ± 79.442*
Pentose phosphate pathway		
6-Phosphogluconic acid	50.175 ± 1.396	60.930 ± 1.607*
Ribose 5-phosphate	1.061 ± 0.061	1.104 ± 0.122
UDP-glucose	0.781 ± 0.029	0.644 ± 0.051*
ADP-ribose	0.468 ± 0.021	0.429 ± 0.024
Galactose 1-phosphate	51.098 ± 0.153	46.520 ± 1.665*
Glucose 1-phosphate	2.141 ± 0.789	2.238 ± 1.271
Ribose 1-phosphate	20.622 ± 1.004	26.839 ± 0.737*
Glutathione metabolism		
Glutathione (GSH)	76.586 ± 3.408	158.868 ± 5.457*
Glutathione (GSSG)	1633.081 ± 35.013	1197.879 ± 26.676*
* S*-Adenosylhomocysteine	10.261 ± 0.119	12.041 ± 0.695
Cystathionine	35.019 ± 1.206	27.087 ± 1.050*
Homoserine	3.362 ± 0.726	6.506 ± 1.732
Threonine	4798.143 ± 132.978	5719.924 ± 160.113*
Carnitine and choline metabolism		
Carnitine	300.006 ± 5.024	327.691 ± 1.952*
Choline	2150.539 ± 113.030	2923.430 ± 123.61*
Betaine aldehyde	413.371 ± 8.955	475.428 ± 9.235*
Betaine	764.813 ± 8.778	776.519 ± 15.539
Folic acid	0.238 ± 0.022	0.197 ± 0.008*

*P < 0.05.

^a^Values are the means of three replicates for the peak area relative to the internal standard ± standard deviation (SD).

^b^High-fat diet 45%.

^c^High-fat diet 45% + cyanidin-3-*O*-β–D-glucoside oral administration.

C3G reduced fasting plasma glucose concentrations (Fig. 2a) but increased the levels of metabolites in the first phase of glycolysis including glucose 6-phosphate and fructose 6-phosphate (Table 1). These results suggest that C3G elevated hepatic glucose uptake by metabolic trapping. C3G indeed increased glucose uptake in HepG2 cell and C2C12 myotubes (Fig. 2g). Although fructose 1,6-bisphosphate, a product of phosphofructokinase-1, was unaltered, the levels of metabolites in the second phase of glycolysis including pyruvic acid and lactic acid levels were substantially reduced in the C3G group (Table 1), which suggest decreased rate of glycolysis by C3G was independent of phosphofructokinase-1 activity. The 6-phosphoglucuronate levels were increased in the liver in the C3G group (Table 1), and hepatic glycogen concentrations were not different between the control and C3G groups (Fig. 2c). These results suggest that glucose 6-phosphate may be directed to the pentose phosphate pathway or other pathways than to glycolysis and glycogen synthesis in C3G fed livers.

Acetyl CoA levels were unaltered, but the level of citrate was dramatically reduced and nearly undetectable in the C3G group compared with the controls (Table 1). Citrate, the first metabolite in the citric acid cycle, is an activator of the anabolic pathway and an inhibitor of the catabolic pathway of fatty acid and glucose metabolism^30^. The concentration of malonyl-CoA, an inhibitor of mitochondrial fatty acid oxidation^31^, was reduced substantially following C3G administration (Fig. 3b). Furthermore, the level of β-hydroxybutyrate was substantially increased by C3G (Fig. 3b). By immunoblotting, pyruvate dehydrogenase kinase 4 (PDK4) expression and PDK4-mediated phosphorylation of pyruvate dehydrogenase complex (PDH, ^pSer293^E1α subunit) were increased by C3G administration (Fig. 3c). Glycerol 3-phosphate was increased in C3G-treated livers (Table 1). Glycerol 3-phosphate can be produced from dihydroxyacetone phosphate by glycerol 3-phosphate dehydrogenase or from the transported lipolyzed glycerol in adipose tissue^32^. These results suggest that C3G suppressed fatty acid synthesis but increased catabolic pathways including fatty acid oxidation, ketogenesis, and at least in part increased lipolysis in white adipose tissues.

The levels of carnitine and its related metabolites choline and betaine aldehyde were increased in the livers of C3G-treated mice compared with those of control mice (Table 1). Carnitine is critical in mitochondrial fatty acid transport and subsequent fatty acid oxidation; thus, increased levels in the blood after carnitine administration have been shown to reduce body weight and body mass index^33^. Thus, elevated levels of carnitine metabolites could contribute to increase fatty acid oxidation and body fat reduction. The levels of α-ketoglutarate, fumarate, and malic acid were reduced but succinate levels were increased in the C3G group compared with the control group (Table 1).

In addition, C3G increased the levels of amino acids, particularly, branched amino acids, compared with those in HFD-fed control mice (Supplementary Table 2). These findings indicate that C3G induces cellular protein degradation pathways, such as autophagy. Branched amino acids have diverse roles and it has been reported that branched chain amino acid could protect hepatic steatosis and NAFLD, thus increased branched chain amino acids may reinforce improvement of NAFLD in mice^34^.

The level of reduced glutathione (GSH) and the ratio of reduced GSH to oxidized GSSH were decreased in the livers of C3G-treated mice (Table 1), which implies that C3G increased antioxidant capacity in the liver. As above mentioned, C3G increased glucose 6-phosphate conversion to 6-phosphogluconate in pentose phosphate pathway. NADPH produced from pentose phosphate pathway did not induce but decreased fatty acid synthesis. These findings suggest that pentose phosphate pathway may be mildly increased, and the produced NADPH may be utilized to increase GSH levels antioxidant capacity in the liver.

The results of the metabolomic analysis collectively indicated that hepatic glucose uptake was increased by C3G and that conversion of glucose 6-phosphate to the pentose phosphate pathway or other pathways was increased. Most importantly, C3G stimulates catabolic energy metabolism including hepatic fatty acid oxidation, ketogenesis, and possibly white adipose lipolysis, which indicates a shifting energy metabolism towards the fat-consuming mode.

### C3G binds and activates PPARs

**Fig. 4:**
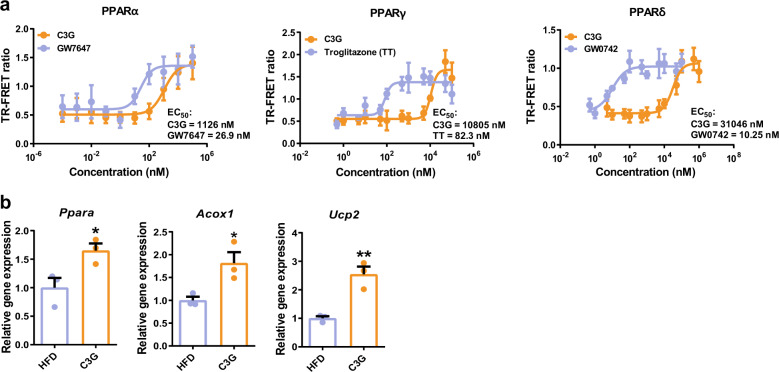
C3G activates PPARs by increasing coactivator recruitment. **a** TR-FRET assay of PPAR subtypes with C3G. **b** PPAR and its target gene expression in control and C3G mice. *P < 0.05 and **P < 0.01 compared with controls.

C3G activates PPARs, as it has been reported that C3G induces PPAR gene expression and has hypolipidaemic and antiobesogenic effects^35^. C3G also ameliorated hypertriglyceridaemia and NAFLD and increased the rate of fatty acid oxidation (Fig. 1). Hepatic metabolomic analysis suggested induction of fatty acid oxidation and ketogenesis by C3G, with decreased malonyl-CoA and citrate levels (Table 1 and Fig. 3b). These phenotypic and metabolomic profiles suggested PPAR activation. Accordingly, we investigated whether C3G is a ligand of PPARs. First, the interaction of C3G with the ligand-binding domain (LBD) of PPARs was quantified with coactivator recruitment assays. GW7647, troglitazone, and GW0742 were PPARα, -γ, and -δ/β agonists and the agonist interacted with the PPAR isoforms, respectively. C3G was found to interact with the LBD of three PPAR isoforms and exhibited the highest binding affinity for PPARα. The EC_50_ values of C3G were 1.1, 10.8, and 31.1 µM for the PPARα, -γ, and -δ/β subtypes, respectively (Fig. 4a and Supplementary Table 3). Second, surface plasmon resonance experiments demonstrated that C3G directly interacted with three PPAR subtypes and exhibited the highest affinity for PPARα (Supplementary Fig. 2a and Supplementary Table 3). By qPCR analysis, the mRNA expression of PPARα and its target genes, *Acox and Ucp2*, was shown to be induced in the mouse livers of C3G group (Fig. 4b). Two additional dietary anthocyanins-pelargonidin—3-*O-*glucoside and delphinidin-3-*O*-glucoside—were also shown to interact directly with PPAR LBDs by surface plasmon resonance experiments (Supplementary Fig. 2b). These results demonstrate that agonistic PPARα and -γ activity is not limited to C3G but extends to other anthocyanins; thus, pelargonidin-3-*O*-glucoside and delphinidin-3-*O*-glucoside may exert hypolipidaemic and antiobesogenic effects similar to those of C3G.

In addition, the gene expression of PPARγ and its target genes are also induced in HepG2 and C2C12 myotubes (Supplementary Fig. 3). Previous pharmacokinetic studies reported that the C_max_ of C3G is 0.14–14 µM^11,36–38^, suggesting that dietary C3G activates the PPARα and PPARγ subtypes in vivo. These results demonstrate that PPARα may be a major and direct target protein of C3G during its regulation of hyperlipidaemia and insulin resistance and it is also possible that C3G also activates PPARγ, which further contributes to the improvement of lipid and glucose metabolism.

**Fig. 5:**
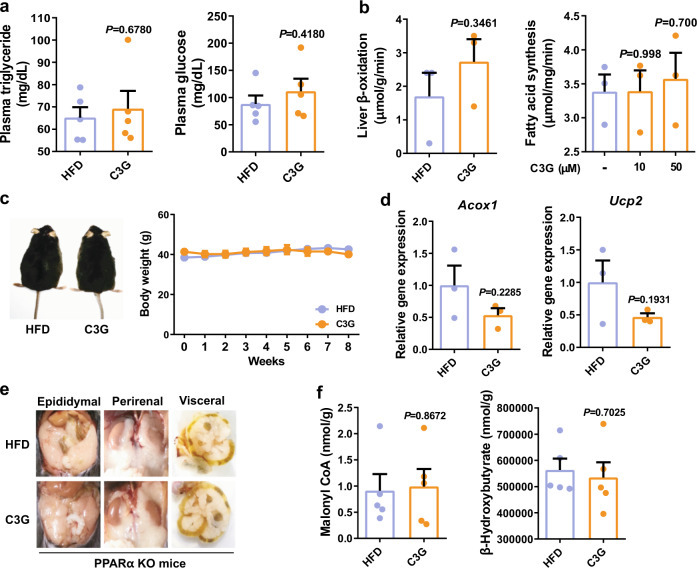
The hypotriglyceridaemic, hypoglycaemic, and antiobesogenic effects of C3G are negated in PPARα-deficient mice. **a** Fasting plasma triglyceride and glucose concentrations. **b** The rates of fatty acid oxidation and synthesis in mouse liver tissues. Fatty acid oxidation in the livers was assessed using livers of control and C3G mice while fatty acid synthesis in the livers was measured in liver homogenates treated with vehicle (double distilled water) or two concentrations of C3G. **c** Changes in body weight and adipose tissue images in mice fed on HFD and orally administered C3G. **d** Fatty oxidation gene expression in HFD-fed mice administered C3G. **e** White adipose tissue in PPARα-deficient mice. **f** The levels of hepatic malonyl-CoA and β-hydroxybutyrate.

Next, we performed feeding studies in PPARα-deficient mice orally administered C3G for 8 weeks. Reductions in plasma TG and fasting glucose concentrations were completely abrogated in PPARα-deficient mice (Fig. 5a). Changes in the rates of fatty acid synthesis and oxidation, body weight, fat mass, white-to-brown adipose tissue weight, mRNA expression levels of the PPARα responsive genes, *Acox1* and *Ucp2*, malonyl-CoA, and ketone bodies in the liver were also nullified in PPARα-deficient mice (Fig. 5b–f and Supplementary Table 4). These results demonstrated that the hypolipidaemic, hypoglycaemic, and antiobesogenic effects of C3G are primarily dependent on PPARα activation.

### C3G reduces adiposity with increases energy expenditure

**Fig. 6:**
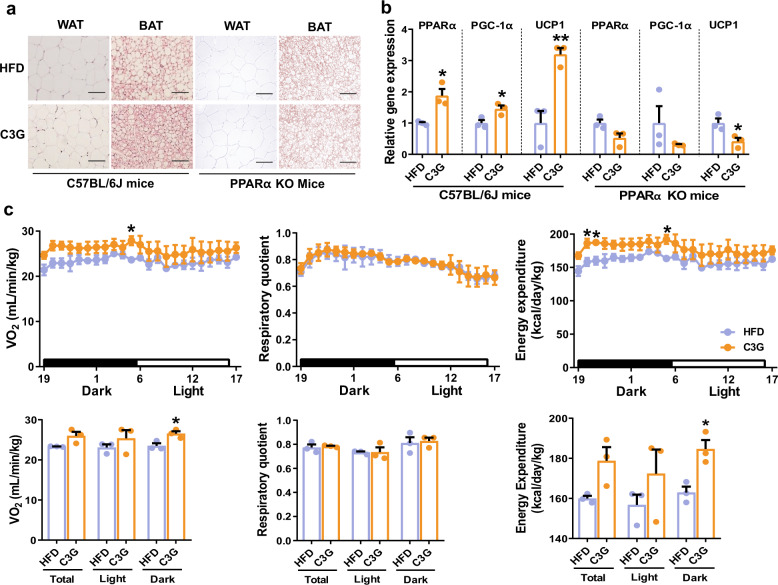
C3G induces oxygen consumption and energy expenditure in HFD-fed mice administered C3G. **a** H&E staining of white adipose tissue (WAT) and brown adipose tissues (BAT) in wild-type (C57BL/6 J) and PPARα-knockout (KO) mice. Scale Bar: 50 µm **b** Thermogenic gene expression in BAT of wild-type and PPAR KO mice. **c** Indirect calorimetric analysis of HFD-fed mice that were administered C3G. *P < 0.05 and **P < 0.01 compared with controls.

Metabolome analysis revealed increased oxidative metabolism induced by C3G. C3G also reduced adiposity in HFD-fed mice, and the antiobesogenic mechanism of C3G was further investigated in mice. C3G administration for 8 weeks reduced visceral adipose tissue and adipocyte size, while the reduction in white adipose tissues induced by C3G was abolished in PPARα-deficient mice. Cells in the brown adipose tissue of HFD-fed mice administered C3G were smaller (Fig. 6a), and the mRNA expression levels of PPARα, PGC-1α and UCP1 in brown adipose tissue were higher; however, these C3G-induced changes were not observed in PPARα-deficient mice (Fig. 6b). Activation of the PPARα-PGC-1α-UCP1 signalling axis by C3G in brown adipose tissue increased oxygen consumption and energy expenditure and caused adiposity in vivo.

To investigate the effects of C3G on respiratory metabolism, indirect calorimetry was performed in wild-type mice orally administered C3G for 2 weeks. Compared with the vehicle control treatment, C3G increased oxygen consumption and energy expenditure, especially in the dark cycle (Fig. 6c). These results demonstrated that C3G reduces body fat accumulation via increased mitochondrial oxidative metabolism and thermogenesis in brown adipose tissue and energy expenditure via the activation of PPARs. The concentrations of malonyl-CoA and β-hydroxybutyrate in the liver were not different between PPARα-deficient mice and control mice (Fig. 5f). These results collectively demonstrate that C3G reduces adiposity in mice by inducing hepatic fatty acid oxidation and brown adipocyte thermogenesis in a PPARα-dependent manner. These results demonstrate that PPARα is a major target protein for C3G in the regulation of energy metabolism.

## Discussion

Several reports have also revealed the diverse biological activities of cyanidin aglycone and C3G, including the regulation of lipid and glucose metabolism, oxidative stress, and inflammation^1,18,19^; however, none of these studies have clearly demonstrated the direct molecular targets of C3G. The oral administration of C3G to HFD-fed mice for 8 weeks reduced hepatic and plasma TG concentrations and induced hepatic fatty acid oxidation, increased brown adipocyte activity, oxygen consumption, and energy expenditure, but these effects of C3G were reversed in PPARα-deficient mice.

**Fig. 7:**
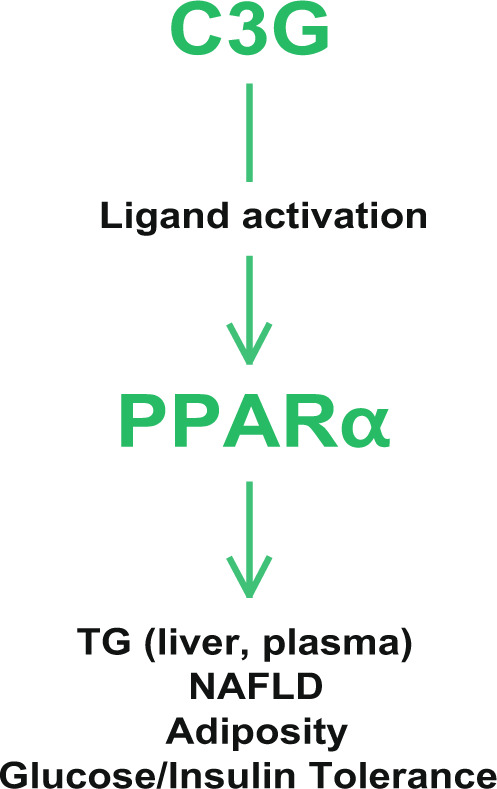
Mechanism by which C3G regulates lipid and glucose metabolism. C3G directly interacts with PPARs with the highest affinity to PPARα to increase hepatic fatty acid oxidation and reduce fatty acid synthesis. These effects lead to reduced plasma and hepatic TG concentrations, with increased ketogenesis, improves NAFLD, glucose and insulin tolerance and adiposity.

We demonstrated that C3G directly interacts with the LBD of PPARs and exhibits the highest affinity for the PPARα subtype of PPARs. To the best of our knowledge, this study is the first to describe a direct interaction between C3G and PPARs. Results showed that PPARγ gene expression was also induced by C3G. However, whether C3G activates PPARα alone or induces both PPARα and -γ, the metabolic effects of C3G were abrogated in PPARα KO mice, which demonstrate activation of PPARα is a key for C3G activity. PPARγ activation, if occurs by C3G in vivo, may reinforce the metabolic effects. The binding affinity of PPARγ to C3G is ~10 times weaker than the affinity to PPARα as demonstrated in TR-FRET and SPR experiments in our study.These results collectively demonstrated that PPARα may be a primary target protein of the metabolic effects of C3G (Fig. 7).

Dietary C3G is absorbed by glucose transporters in the intestinal epithelium and delivered to the circulation to carry out biological activities in different tissues, including the liver, skeletal muscle, and adipose tissue. Membrane transport of C3G in humans has not been clearly elucidated; however, the cellular uptake of anthocyanins, including C3G, is reduced by inhibitors of bilitranslocase^39,40^, glucose transporters^41^, breast cancer resistance protein^42^, and multidrug resistance-1^42^, which suggests that multiple transporters with broad substrate specificity may be involved in anthocyanin uptake. In aqueous solution, C3G is more stable than cyanidin aglycone in aqueous solution^12^; thus, C3G is the major bioavailable form of cyanidin in the circulation. However, most of the biological effects of cyanidin have been studied using cyanidin aglycone rather than C3G^43^.

The levels of C3G in plasma and different tissues were vary from 0.14 to 14 µM depending on the amount of oral intake, type of analysis methods, and animal models^11,36–38^. Isotope labeled C3G feeding studies showed bioavailability of C3G is approximately 12.4%^11^. Consumption of a 500 mg oral bolus dose of ^13^C-labelled C3G showed that C_max_ was 0.14 and 0.334 µM in plasma and urine, respectively^36^. On the other hand, a pharmacokinetic study of anthocyanin-rich extract from wild mulberry, composed of 79% of C3G, showed that C_max_ was 5.7 µg/mL (11.8 µM) in plasma^37^. More recently, in a study by Chen Y et al., oral gavage of 50 mg/kg of C3G in SD rats showed that C_max_ of C3G was 14 µM at 45 min^38^. In a systematic review by Sandoval–Ramirez BA et al. reported that anthocyanin concentrations in tissues are 6–217 ng/g tissue weight in different tissues of several animal models and authors concluded that anthocyanin such as C3G may have an important role in human health at this concentration range^44^.

These reports are in-line with our findings that C3G in physiological conditions may exert potent biological activity as a PPARα ligand. Although the plasma levels and tissue levels of C3G is low but it should not be ruled out that local concentration of C3G (e.g. in nucleus or in cytosol) may be high enough to activate PPARα and this possibility should be studied in the future.

Human pharmacokinetic studies have shown that the relative bioavailability of C3G in humans was ~12.4%, the maximum serum concentration was 5.97 μM at 10.25 h, and the half-life ranged between 12.44 and 51.62 h when 500 mg of isotopically labelled C3G was consumed^11^. We measured the EC_50_ of C3G for PPARα (1.1 μM) in this study, and the results indicate that circulating C3G can sufficiently activate PPARα. Cyanidin aglycone is a PPARα agonist that reduces lipid accumulation in cultured hepatocytes^45^; however, C3G is more stable in aqueous solution^12^ and has an EC_50_ value lower than that reported for cyanidin aglycone, and C3G is the major cyanidin form found in the circulation after cyanidin intake; thus, C3G is an effective natural substance that activates PPARα. PPARα activation has metabolic benefits. Thus, potent PPARα agonists, fibrates, have been used to treat hyperlipidaemia, but these agents have side effects including stomach pain and liver problems^46^. However, C3G, a natural substance with a relatively moderate PPARα-binding affinity, may be used to treat or prevent hypertriglyceridaemia. In cultured cells, it is possible that C3G induced PPARγ as well, thus we do not rule of the possibility that C3G activates PPARγ in vivo as well. C3G is metabolized quickly after intestinal absorption; however, studies found that the concentration of C3G is substantially high in vivo.

In the metabolomic analysis, C3G increased metabolites in the first phase of glycolysis (glucose 6-phosphate, fructose 6-phosphae, dihydroxyacetone phosphate) but reduced metabolites in the second phase of glycolysis (3-Phosphoglyceric acid, 2-Phosphoglyceric acid, phosphoenolpyruvic acid, pyruvic acid) as well as lactic acid, a surrogate marker for glycolysis activity. These findings suggest that C3G may increase glucose uptake but reduced glycolysis activity. C3G also reduces levels of metabolites in citric acid cycle (with an exception of succinate) including citric acid, an allosteric activator for anabolism. These findings suggest that C3G suppressed anabolic metabolism, which was confirmed by reduced fatty acid synthesis rates measured in livers of mice and HepG2 hepatocytes.

In addition, the levels of 6-phosphogluconate was increased but not ribose 5-phosphate and ribose 1-phosphate, which are surrogate markers of ribulose 5-phosphate, a final product in the oxidative phase of pentose phosphate pathway. These suggest that C3G only mildly increase conversion of glucose 6-phosphate to pentose phosphate pathway. pentose phosphate pathway increases NADPH production. NADP is a reducing power required in biosynthetic pathways but also is important in several other metabolism including glutathione metabolism. In our results, the level of reduced glutathione (GSH) was increased but oxidized form (GSSG) was decreased. Thus, it is possible that NADPH produced from pentose phosphate pathway may be consumed to maintain cellular oxidative capacity of liver increasing the levels of GSH.

Increased phosphorylation of PDH by PDK4, increased ketone body levels and dramatically reduced hepatic citrate and malonyl-CoA levels suggested that C3G stimulated fatty acid oxidation. Citrate is an activator of lipid synthesis while malonyl-CoA inhibits CPR-1 to suppress acyl transport to mitochondrial matrix in fatty acid. PDH is an enzyme that converts pyruvate to acetyl CoA, and its activity is largely inhibited by PDK4^47^. ^pSer293^E1α subunit of PDH is the most rapidly phosphorylated site by PDKs including PDK4 and PDH inhibition by PDK4 increases acetyl CoA flux to the citric acid cycle from fatty acid oxidation while suppressing production of acetyl CoA from pyruvate and glycolysis, thus reducing fat accumulation in extra-adipose tissue, ameliorating lipotoxicity, and improving insulin resistance^35^.

Branched chain amino acids are functional amino acids that can directly converted into energy fuels and are known to improve muscle functions. Although the significance of hepatic branched chain amino acids is not clear yet, there is a study that reported protective role of branched chain amino acids in the prevention of NAFLD/NASH^34^ and we suggest that increased branched chain amino acids in the mouse liver may further contribute to the improved NAFLD by C3G.

The changes in betaine, choline, and carnitine levels in liver tissues following C3G administration demonstrated that C3G activates fatty acid oxidation and decreases plasma and hepatic TG levels. C3G reduced lipid accumulation in brown adipose tissue, with induction of oxygen consumption and energy expenditure measured by indirect calorimetry. These metabolic alterations could be the result of PPAR activation; however, these effects were negated in PPARα-deficient mice. These findings suggest that PPARα is a major target protein for C3G in the regulation of energy metabolism.

In conclusion, in this study, we demonstrate that the molecular targets of the small molecule C3G are PPARs and that C3G induces glucose and fatty acid catabolism to improve glucose tolerance and hepatic steatosis in HFD-fed mice. Increased thermogenic gene expression in brown adipose tissue, and increased energy expenditure collectively caused the metabolic alterations induced by C3G.

## Methods

### Materials and reagents

Commercially available materials and reagents are listed in Supplementary Table 5.

#### Cell culture and mouse experiments

HepG2 cells and HEK293 cells were obtained from the Korean Cell Line Bank (Seoul, Korea) and cultured and maintained in Dulbecco’s modified Eagle’s medium (DMEM) with 10% foetal bovine serum (FBS) and 1% penicillin/streptomycin at 37 °C with a humidified atmosphere of 5% CO_2_. Stock of C3G and fenofibrate were prepared in ddH_2_O and DMSO, respectively, and used for the treatment of the cells. HepG2 cells were seeded at a density of 10^6^ cells/well in 6-well plates for 24 h and then cells were lipid-loaded with free fatty acids (400 μM of palmitic acid and 400 μM of oleic acid) with 0.5% bovine serum albumin (GenDEPOT, TX, USA) for 24 h. Lipid-loaded cells were then treated with C3G (10 and 50 μM) or GW7647 (1 μM) as the positive control for lipid analysis. For mouse experiments, C57BL/6 J male mice were purchased from Samtako Co. (Kyunggido, Korea), and PPARα-deficient mice were purchased from Taconic (Hudson, NY, USA). Male mice were used in the experiments. C57BL/6 J and PPARα-deficient male mice were maintained under a 12-h light/12-h dark cycle at a temperature of 21–25 °C and a relative humidity of 50–60% and fed with a purchased 45% HFD (45% of calories from fats; Central Lab. Animal Inc., Seoul, Korea) for 4 weeks. Then, the mice were randomly assigned to two groups: HFD-fed with vehicle (PBS) and HFD-fed with oral administration of C3G (50 mg/kg body weight) for an additional 8 weeks. Body weight was assessed weekly. At the end of the experimental period, the mice were fasted for 12 h and sacrificed. Blood was collected in EDTA tubes (BD Biosciences, CA, USA) retro-orbitally by cardiac puncture, centrifuged for 30 min at 2000 rpm at 4 °C to collect plasma samples, and stored at −80 °C. The organs, including the liver, brown adipose tissues, and white adipose tissues (epididymal, visceral, and perirectal fat), were collected and snap-frozen in liquid nitrogen and then stored at −80 °C for further use. All animal experiments were performed according to a protocol approved by the Animal Experiment Committee of Korea University (Protocol No. KUIACUC-20090420-4).

#### Metabolomic analysis

Sample preparation and detailed methods for CE-MS and GC-TOF-MS are presented in the Supplementary Methods.

#### Statistics and reproducibility

All of the data are shown as the means ± SEMs, and one-way analysis of variance (ANOVA) was used to calculate the significance of the difference between each set of two groups. A value of *P* < 0.05 was considered significant. Statistical analysis was performed with GraphPad Prism 8.0 (GraphPad Software, Inc., San Diego, Ca, USA). For metabolomic analysis, ANOVA was performed using SPSS (Version 12.0, Chicago, IL, USA) to assess statistically significant differences in metabolites in plasma samples from mice fed C3G with a HFD. Duncan’s multirange test was used when the level of significance was set at *P* < 0.05. Multivariate statistical analyses, such as principal component analysis (PCA) and PLSR, were performed with SIMCA-P+(Version 11.0 Umetrics, Umeå, Sweden). PCA and PLSR were used to distinguish different diets on the basis of the content of metabolites in samples and to explore the correlations between metabolites and obesity-related biochemical parameters.

### Reporting summary

Further information on research design is available in the Nature Research Reporting Summary linked to this article.

## Supplementary information

Supplementary Information

Description of Additional Supplementary Files

Supplementary Data 1

Reporting Summary

## Data Availability

Source data underlying plots shown in figures are available in Supplementary Data 1. Full blots are shown in Supplementary Information. Additional data related to this paper are available from the corresponding author on reasonable request.
