# Supplementary material for: A dietary anthocyanin cyanidin-3-O-glucoside binds to PPARs to regulate glucose metabolism and insulin sensitivity in mice
Source: Commun Biol. 2020 Sep 18;3:514. doi: 10.1038/s42003-020-01231-6 (PMC7501857; doi:10.1038/s42003-020-01231-6)
Supplement: Supplementary file 1 — Supplementary Information [file 42003_2020_1231_MOESM1_ESM.docx]

**Supplementary Information**

**A dietary anthocyanin cyanidin-3-*O*-glucoside binds to PPARs to regulate glucose metabolism and insulin sensitivity in mice**

Supplementary Figures 1-3

Supplementary Data - Immunoblot images

Supplementary Tables 1-6

Supplementary Methods

**Supplementary Figures**


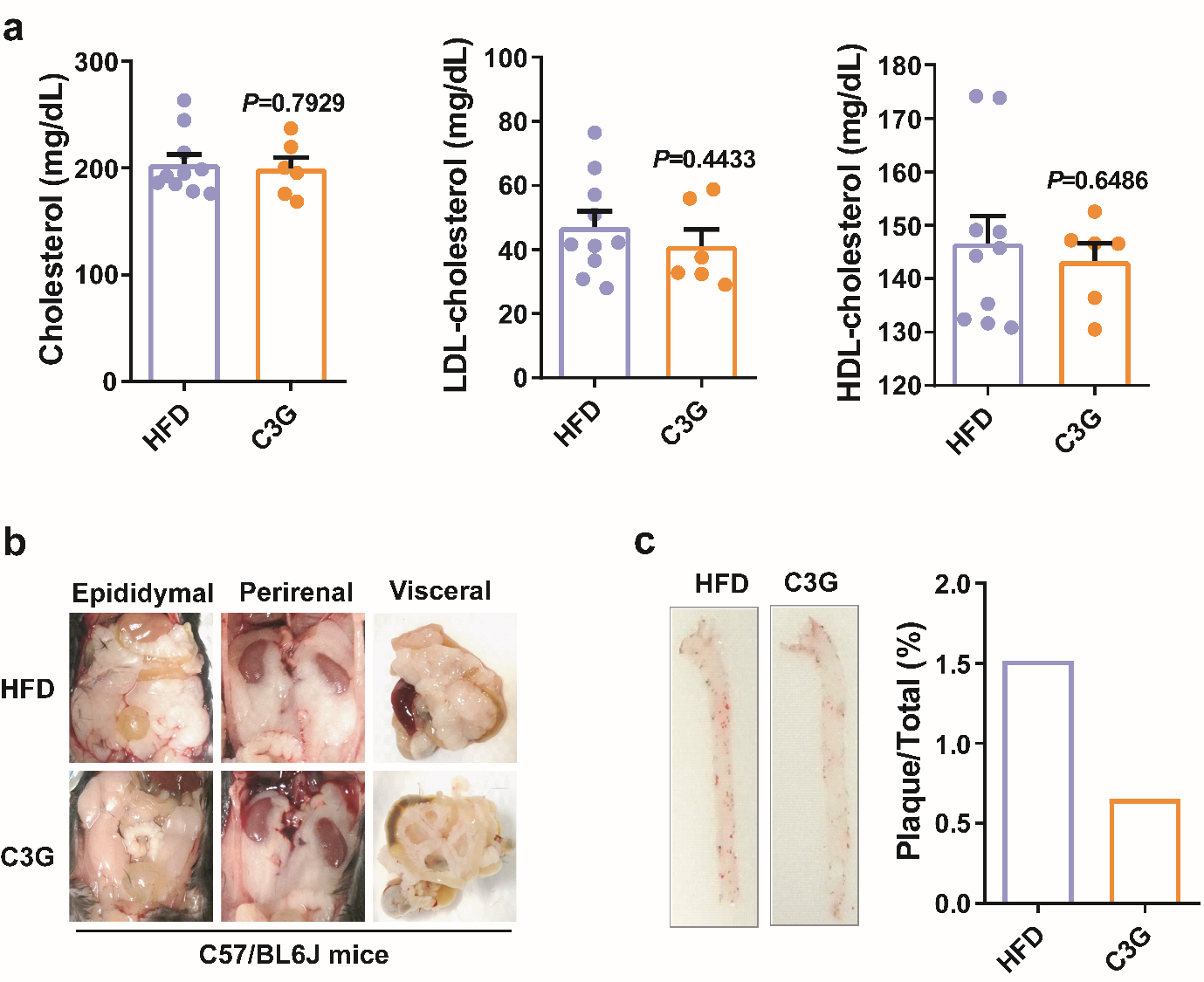


**Supplementary Figure 1.** Effect of C3G on plasma cholesterol levels, adipose tissue, and aortic atherosclerosis of mice fed a HFD. Data are expressed as mean ± SEM (n = 10, HFD; n = 7, C3G).


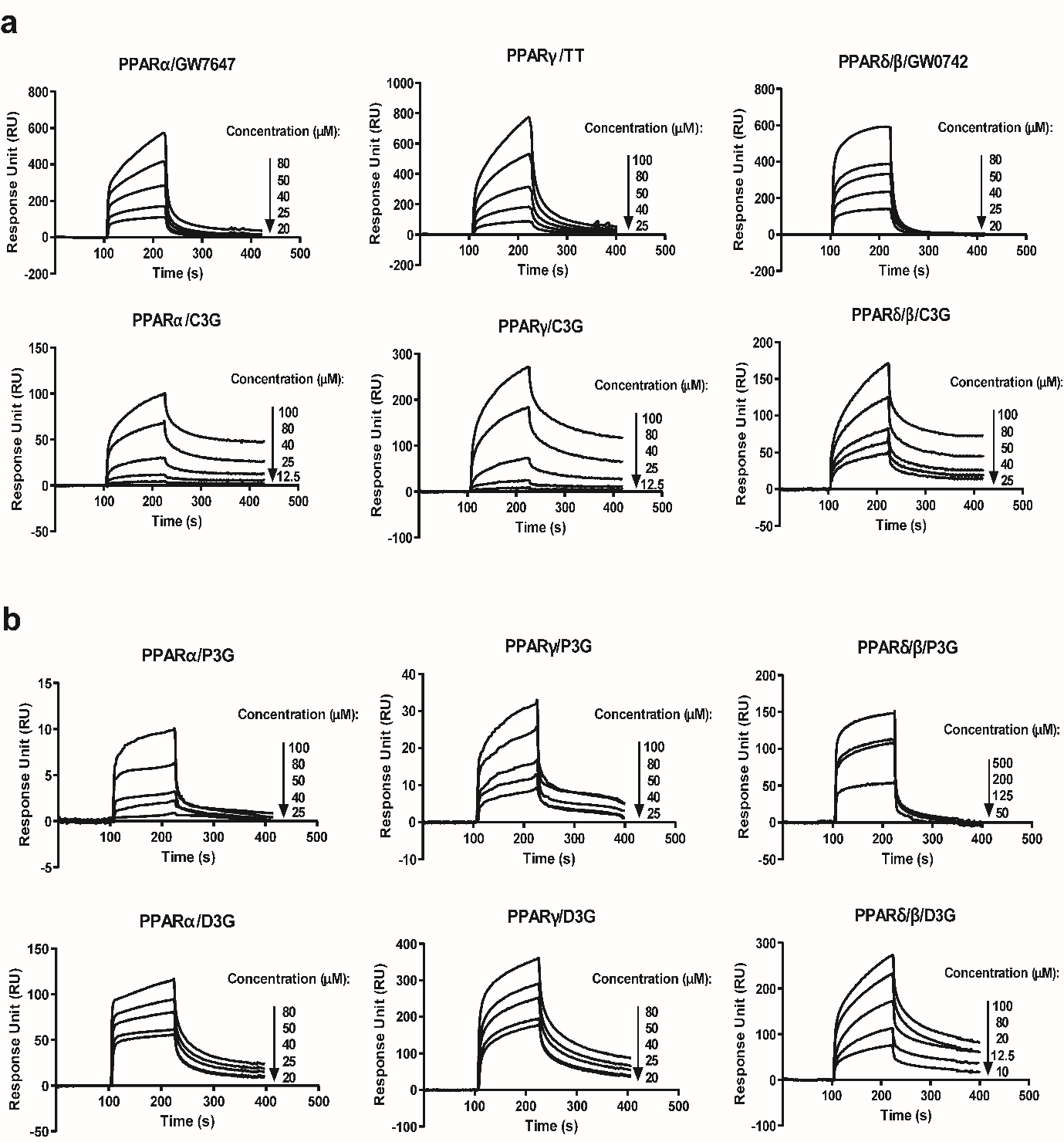


**Supplementary Figure 2.** Surface plasmon resonance (SPR) analysis of C3G and other anthocyanin glycosides. A. SPR analysis of C3G compared with PPAR agonists. B. SPR analysis of delphinidine-3-O-glycoside (D3G) and peonidine-3-o-glycoside (P3G) compared with PPAR agonists.


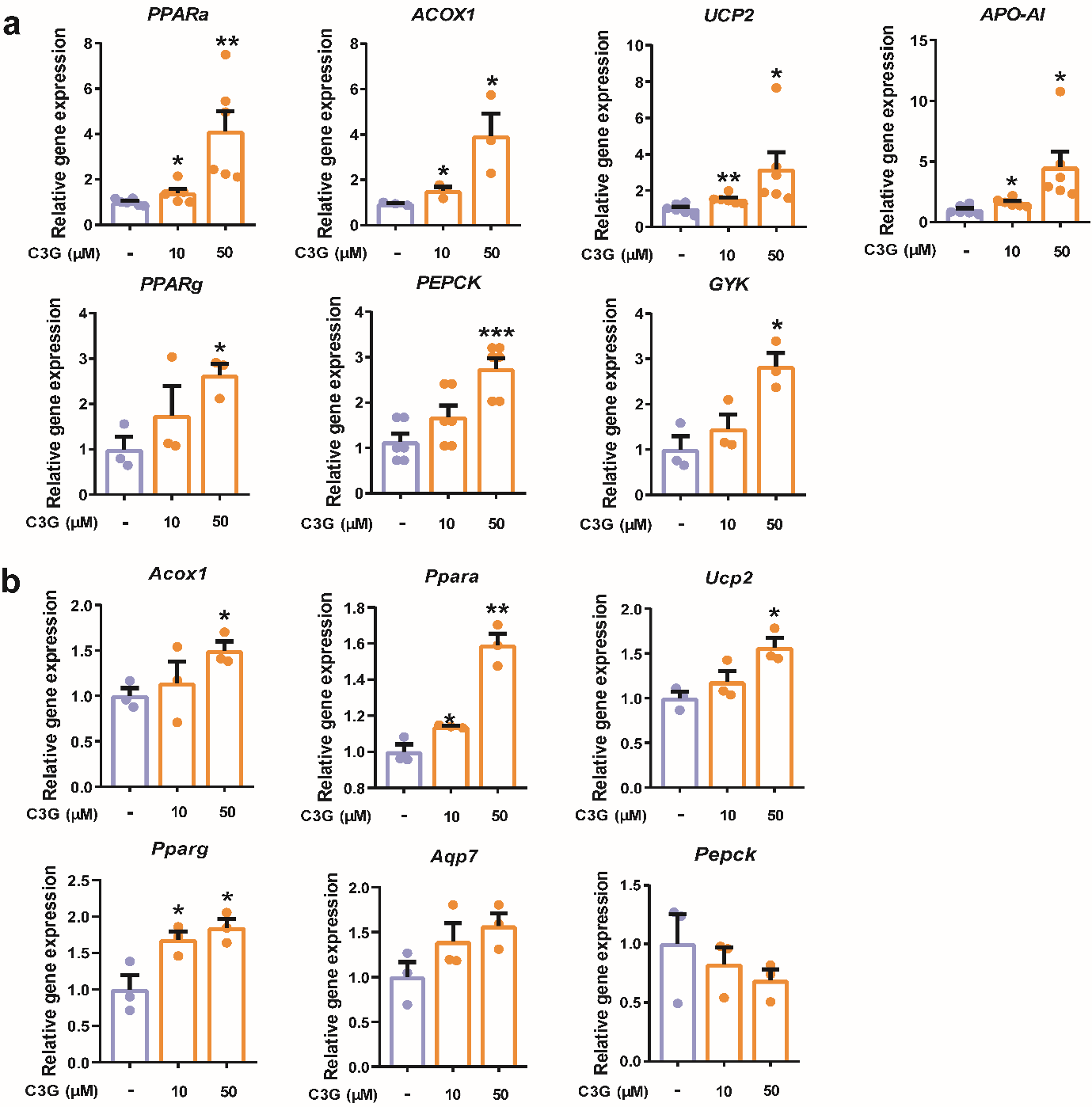


**Supplementary Figure 3.** The expression of PPARα and γ and their specific target gene expressions in HepG2 cells and C2C12 myotubes. A. Gene expressions in HepG2 cells. B. Gene expressions in C2C12 myotubes. **P* < 0.05, ***P* < 0.01, and ****P* < 0.005 compared with controls.

**Supplementary Figure 4.** Full blots Shown in Fig. 3C


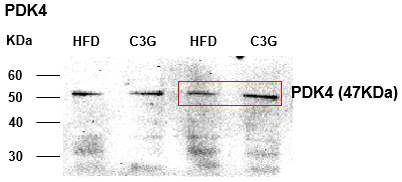

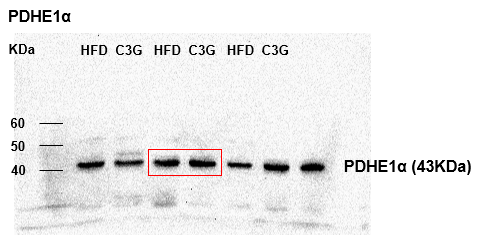


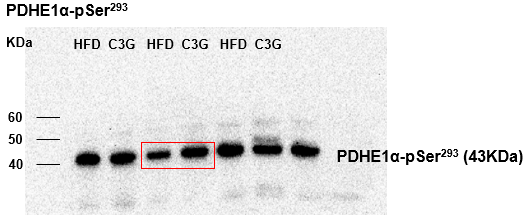


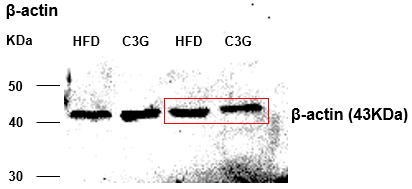


**Supplementary Table 1.** Adipose tissues, skeletal muscle, and liver weights in HFD mice with C3G administration.

|  | HFD | | | C3G | | |
| --- | --- | --- | --- | --- | --- | --- |
| Epididymal Fat (g) | 2.45 | ± | 0.16 | 2.41 | ± | 0.19 |
| Visceral Fat (g) | 1.67 | ± | 0.11 | 0.98 | ± | 0.19^⁎^ |
| Perirenal Fat (g) | 1.52 | ± | 0.10 | 1.19 | ± | 0.15 |
| Total White Adipose Tissue (WAT, g) | 5.63 | ± | 0.20 | 4.58 | ± | 0.52 |
| Brown Adipose Tissue (BAT, g) | 0.29 | ± | 0.03 | 0.36 | ± | 0.04 |
| WAT/BAT | 20.81 | ± | 2.07 | 12.90 | ± | 0.87^⁎^ |
| Skeletal Muscle (g) | 0.68 | ± | 0.04 | 0.76 | ± | 0.06 |
| WAT/Skeletal Muscle | 8.43 | ± | 0.49 | 5.85 | ± | 0.80^⁎^ |
| Liver (g) | 1.59 | ± | 0.13 | 1.37 | ± | 0.17 |
| Liver/Body weight | 0.036 | ± | 0.002 | 0.034 | ± | 0.003 |

Data are expressed as mean ± SEM (n = 10, HFD; n = 7, C3G).

* *P* < 0.05

**Supplementary Table 2.** Identification of amino acids in liver tissues from mice fed a HFD and C3G for 8 weeks using GC-TOF-MS.

| Amino acids | HFD (nmol/g) | C3G (nmol/g) |
| --- | --- | --- |
| Non-polar amino acids |  |  |
| Gly | 8387 ± 257 | 10032 ± 232^⁎⁎^ |
| Ala | 12598 ± 402 | 12811 ± 344 |
| Met | 2112 ± 79 | 2648 ± 69^⁎⁎⁎^ |
| Branched-chain amino acids |  |  |
| Val | 4657 ±194 | 5705 ±193^⁎⁎^ |
| Ile | 2562 ± 27 | 3132 ± 122^⁎^ |
| Leu | 4914 ± 166 | 6097 ± 216^⁎⁎^ |
| Polar amino acids |  |  |
| Ser | 6846 ± 163 | 7863 ± 173^⁎⁎^ |
| Thr | 4798 ± 133 | 5720 ± 160^⁎⁎^ |
| Cys | 10 ± 2 | 31 ± 6^⁎^ |
| Pro | 4156 ± 131 | 5083 ± 159^⁎⁎^ |
| Asn | 543 ± 10 | 646 ± 7^⁎⁎⁎^ |
| Gln | 1126 ± 43 | 1159 ± 23 |
| Charged amino acids |  |  |
| Lys | 5943 ± 208 | 7198 ± 229^⁎⁎^ |
| Arg | 11 ± 1 | 8 ± 0^⁎^ |
| His | 1398 ± 50 | 1571 ± 38^⁎^ |
| Asp | 5681 ± 157 | 7147 ± 198^⁎⁎⁎^ |
| Glu | 8054 ± 252 | 9034 ± 199^⁎⁎^ |
| Aromatic amino acids |  |  |
| Phe | 2751 ± 56 | 3411 ± 88^⁎⁎⁎^ |
| Tyr | 2181 ± 55 | 2937 ± 68^⁎⁎⁎^ |
| Trp | 457 ± 7 | 585 ± 3^⁎⁎⁎^ |

^1^Values are the means of three replicates for the peak area relative to the internal standard ± standard deviation (SD). * *P* < 0.05; **, *P* < 0.01; ***, *P* < 0.005

|  | C3G | | | GW7647 | Troglitazone | GW0742 |
| --- | --- | --- | --- | --- | --- | --- |
|  | PPARα | PPARγ | PPARδ | PPARα | PPARγ | PPARδ |
| SPR | 456 nM | 1.36 μM | 4.96 μM | 13.2 nM | 377 nM | 102 nM |
| TR-FRET | 1.13 μM | 10.8 µM | 31.05 μM | 26.9 nM | 82.3 nM | 10.25 nM |

**Supplementary Table 3.** Binding constant (K_D_) and half maximal effective concentrations (EC_50_) of C3G interacting with the ligand-binding domains of PPARs.

**Supplementary Table 4**. Organ weights of PPARα KO mice administered C3G.

|  | HFD | | | C3G | | |
| --- | --- | --- | --- | --- | --- | --- |
| Epididymal Fat (g) | 1.36 | ± | 0.21 | 1.94 | ± | 0.39 |
| Visceral Fat (g) | 0.37 | ± | 0.05 | 0.46 | ± | 0.05 |
| Perirenal Fat (g) | 0.57 | ± | 0.03 | 0.79 | ± | 0.17 |
| Total White Adipose Tissue (WAT, g) | 2.29 | ± | 0.27 | 3.19 | ± | 0.59 |
| Brown Adipose Tissue (BAT, g) | 0.19 | ± | 0.03 | 0.30 | ± | 0.04 |
| WAT/BAT | 12.81 | ± | 1.25 | 10.71 | ± | 1.17 |
| Skeletal Muscle (g) | 0.24 | ± | 0.01 | 0.38 | ± | 0.07 |
| WAT/Skeletal Muscle | 9.95 | ± | 1.66 | 8.71 | ± | 1.54 |
| Liver (g) | 0.76 | ± | 0.11 | 1.00 | ± | 0.18 |
| Liver/Body weight | 0.02 | ± | 0.003 | 0.03 | ± | 0.005 |

Data are expressed as mean ± SEM (n = 5, HFD; n = 4, C3G).

**Supplementary Table 5**. Chemical reagents, antibodies, proteins, cell lines, mice and assay kits used for this investigation.

| **Chemical Reagents** | **Source** | **Catalogue #** |
| --- | --- | --- |
| Dulbecco’s modified Eagle’s medium (DMEM) | HyClone (Logan, UT, USA) | SH30243.01 |
| Foetal bovine serum (FBS) | HyClone (Logan, UT, USA) | SH30084.03 |
| Penicillin/streptomycin (PEST) | Welgene Inc. | SV30010 |
| Cyanidin-3-O-glucoside (C3G) | Tokiwa Phytochemical Co. (Japan) | P21021 |
| Troglitazone (TT) | Sigma Aldrich (St. Louis, MO) | T2573 |
| GW7647 | Cayman Chemical (Michigan, USA) | 10008613 |
| GW0742 | Cayman Chemical (Michigan, USA) | 10006798 |
| RNAiso Plus | Takara (Otsu, Japan) | 9109 |
| Thunderbird^TM^ SYBR^®^ qPCR Mix | Toyobo (Osaka, Japan) | QPS-201 |
| Triglycerides | Cobas111 (Roche, Switzerland) | 46577594 |
| Cholesterol | Cobas111 (Roche, Switzerland) | 04718917 |
| Aspartate aminotransferase | Cobas111 (Roche, Switzerland) | 04657543 |
| Alanine aminotransferase | Cobas111 (Roche, Switzerland) | 04718569 |
| HDL-cholesterol | Cobas111 (Roche, Switzerland) | 07528604 |
| LDL-cholesterol | Cobas111 (Roche, Switzerland) | 07005806 |
| Control siRNA | Santa Cruz (CA, USA) | sc-37007 |
| **Antibodies** | **Source** | **Catalogue #** |
| PDK4 | Santa Cruz (CA, USA) | sc-14495 |
| PDH1α | Abcam (Cambridge, UK) | ab110330 |
| p-PDH1α | Abcam (Cambridge, UK) | ab92696 |
| β-Actin | Santa Cruz (CA, USA) | sc-47778 |
| Secondary antibodies (anti-mouse) | Santa Cruz (CA, USA) | 31430 |
| **Cell Lines** | **Source** | **Catalogue #** |
| HepG2 (Human hepatocellular carcinoma) | Korean Cell Line Bank | 88065 |
| C2C12 cells (Mouse myoblast) | ATCC | 30-2002 |
| **Mice** | **Source** | **Catalogue #** |
| C57BL/6J | Samtako Co. (Kyunggido, Korea) | C57BL/6JTacSam |
| PPARα-deficient | Taconic (Hudson, NY, USA) | 1640 |
| **Assay Kits** | **Source** | **Catalogue #** |
| Adiponectin | Abcam (Cambridge, UK) | ab108785 |
| Insulin | Alpco (NH, USA) | 80-INSMSU-E01 |
| Malonyl-CoA | Cusabio Biotech Co. (TX, USA) | CSB-E12896m |
| β-Hydroxybutyrate | Cayman (MI, USA) | 700190 |
| LanthaScreen^TM^ TR-FRET | Invitrogen (Carlsbad, CA, USA) | A15878 |
| FGF21 | Merck Millipore | EZRMFGF21-26K |
| Glycogen | Abcam (Cambridge, UK) | ab169558 |

**Supplementary Table 6**. Sequence of primer.

| Gene | Sequence (5’ – 3’) | |
| --- | --- | --- |
|  | Forward | Reverse |
| m*PPARα* | ACCTTGTGTATGGCCGAGAA | AAGGAGGACAGCATCGTGAA |
| m*Acox1* | CACGCAATAGTTCTGGCTCA | GGTCCCATACGTCAGCTTGT |
| m*UCP2* | GCGTTCTGGGTACCATCCTA | GCTCTAAGCCCTTGGTGTAG |
| m*PGC1α* | ACCCACAGGATCAGAACAAACCCT | TTGGTGTGAGGAGGGTCATCGTTT |
| m*UCP1* | TCTTCTCAGCCGGAGTTTCAGCTT | GCAATGTCACCACAGACCAC |
| m*PPARγ* | TTTTCAAGGGTGCCAGTTTC | AATCCTTGGCCCTCTGAGAT |
| m*Aqp7* | GTCTTCCTGTGGGTCTCAGC | CACCGAGATAGCTGCCAGAG |
| m*Pepck* | CTCGAGATGTGGCCAGGATC | GGGCGAGTCTGTCAGTTCAA |
| m*Cyclophylin* | GGCCGATGACGAGCCC | TGTCTTTGGAACTTTGTCTG |
| m*GAPDH* | AACTTTGGCATTGTGGAAGG | ACACATTGGGGGTAGGAACA |
| h*PPARα* | CTATCATTTGCTGTGGAGATCG | AAGATATCGTCCGGGTGGTT |
| h*Acox1* | CTCCTCTGAGAAGCTGGGATT | TCACCTGAGGCTAGGAGTTCA |
| h*UCP2* | ACTGTGCCCTTACCATGCTC | AGGAGAGGCTCAGAAGGGAG |
| h*ApoAI* | CATTTCTGGCAGCAAGATGA | GCCTTCAAACTGGGACACAT |
| h*PPARγ* | GACCACTCCCACTCCTTTGA | CGACATTCAATTGCCATGAG |
| h*PEPCK* | AACAGGAGGTTCGTGACATTCGGA | TAGCTACTACCCAGTGTTCTGTGG |
| h*GyK* | GGATGCCATGAATCGAGACT | AAATCCTCGGGTTCGAGACT |
| h*GAPDH* | ATGGATGATGATATCGCCGCC | CTCCATGTCGTCCAGTTGGT |

*PPARα*, Peroxisome proliferator-activated receptor alpha; *Acox1*, Acyl-CoA Oxidase 1; *UCP2*, Uncoupling protein 2; *PGC1α*, Peroxisome proliferator-activated receptor gamma coactivator 1-alpha; *UCP1*, Uncoupling protein 1; *PPARγ*, Peroxisome proliferator-activated receptor gamma; *Aqp7*, Aquaporin 7; *Pepck*, Phosphoenolpyruvate carboxykinase; *GAPDH*, Glyceraldehyde 3-phosphate dehydrogenase; *ApoAⅠ*, Apolipoprotein A1; *GyK*, Glycerol Kinase

**Supplementary Methods**

Material and product information is in Supplementary Table 5.

**Quantification of plasma glucose, plasma and hepatic lipids and hormones**

Hepatic lipids were extracted as described previously (Hoang et al., 2011). Livers were homogenized in acetone and centrifuged to collect the lipid fractions, and then the lipid fractions were dried at room temperature and dissolved in 95% ethanol. The triglycerides collected in plasma and extracted from the liver, as well as plasma aspartate aminotransferase, alanine aminotransferase, and fasting glucose concentrations, were quantified with enzymatic methods using an automated clinical chemistry analyser (Cobas111, Roche, Basel, Switzerland), according to the manufacturer’s instructions. Plasma adiponectin (Abcam, Cambridge, UK) and insulin (Alpco, NH, USA) levels were measured by ELISA kits according to the manufacturer’s instructions. The homeostatic model assessment–insulin resistance (HOMA-IR) index was calculated according to the following formula: $HOMA-IR=\frac{Glucose x Insulin}{405}$ (glucose in mass units of mg/dL). The insulin sensitivity index was derived using the inverse of the sum of the logarithms of the fasting insulin and fasting glucose following the formula 1 / (log(fasting insulin µU/mL) + log(fasting glucose mg/dL)).

The hepatic malonyl-CoA concentration was assessed by homogenizing livers in 1× PBS, and after two freeze-thaw cycles, the samples were centrifuged to collect supernatants for the ELISA according to the manufacturer’s instructions (Cusabio Biotech Co., Houston, TX, USA). The hepatic β-hydroxybutyrate contents were quantified using a β-hydroxybutyrate (Ketone Body) Fluorometric Assay Kit (Cayman, MI, USA) according to the manufacturer’s instructions. Briefly, livers were homogenized in cold assay buffer (100 mM Tris-HCl, pH 8.5) with protease inhibitors and then centrifuged to obtain the sample. Test samples were combined with β-HB fluorometric cofactors (NAD^+^), a mixture of lyophilized enzyme, 100 mM Tris-HCl (pH 8.5), and Fluorometric Developer Reagent (WST-1 solution), and the reaction was initiated by adding 3-hydroxybutyrate dehydrogenase, followed by incubation at 37°C for 30 min. The fluorescence was quantified using an excitation wavelength of 530-540 nm and an emission wavelength of 585-595 nm.

**Histological analysis**

The livers and adipose tissues were fixed with 4% paraformaldehyde and then stained with haematoxylin and eosin (H&E) in the Histopathology Department of Anam Korea University Hospital (Seoul, Korea). Adipocyte size was assessed using an Axio Imager M1 microscope (Carl-Zeiss, Oberkochen, Germany).

**Oral glucose tolerance test (OGTT) and insulin tolerance test (ITT)**

After 8 weeks of C3G administration, the mice were fasted overnight and orally administered glucose (1.5 g/kg body weight) for the OGTT or fasted for 4 h and intraperitoneally injected with insulin (0.35 units/kg body weight) for the ITT. The blood glucose concentrations were measured at 0, 15, 30, 60, 90, and 120 min after glucose feeding or insulin injection using a portable glucose meter (Accu-Check Go, Roche).

**Glucose uptake assay**

Glucose uptake was measured using 2-(N-(7-nitrobenz-2-oxa-1,3-diazol-4-yl)amino)-2-deoxy-glucose (2-NBDG; Invitrogen, Carlsbad, CA, USA). C2C12 myoblasts were cultured in DMEM supplemented with 2% horse serum (Sigma-Aldrich, St. Louis, MO, USA) and 1% PEST. After four days of differentiation, C2C12 myotubes were used for glucose uptake. C2C12 myotubes and HepG2 cells were treated with C3G for 24 h and then exposed to fatty acid free-bovine serum albumin (GenDEPOT, Barker, TX, USA)-binding 400 µM of palmitic acid (Sigma) and oleic acid (Junsei, Tokyo, Japan) for 24 h. Next, each cell was washed with Krebs buffer, and 100 nM of insulin (Sigma) was added to each cell, followed by 10 min incubation. Then, 250 µM of 2-NBDG was added to each well and incubated for 15 min in the dark. After each cell was washed twice with ice-cold PBS, the fluorescence intensities of 2-NBDG were detected using SpectraMax i3x microplate reader (Molecular Devices, Sunnyvale, CA, USA) at excitation/emission 465/540 nm.

**Quantitative (q) PCR analysis**

The total RNA from the livers was extracted using RNAiso Plus according to the manufacturer’s instructions, and the cDNA was synthesized using a Rever Tra Ace^®^ qPCR RT Master Mix (Toyobo, Osaka, Japan) according to the manufacturer’s instructions. To synthesize the cDNA, 1 μg of total RNA was mixed with the “4× DN Master Mix” (a buffer solution containing RNase inhibitor) with gDNA remover and then incubated at 37°C for 15 min. The “5× master mix” containing the highly efficient reverse transcriptase “Rever Tra Ace®”, RNase inhibitor, oligo dT primer, random primer and dNTPs was further added to the mixture and incubated at 50°C for 5 min followed by at 98°C for 5 min. The levels of mRNA expression were assessed using the iQ5 Real-Time PCR Detection System (Bio-Rad, Hercules, CA) and THUNDERBIRD^TM^ SYBR^®^ qPCR Mix. Relative levels of gene expression were calculated with the threshold cycle (C_T_) method. The primer sequences used for qPCR are given in Supplementary Table 6.

**Immunoblotting analysis**

The proteins from the livers were extracted, and immunoblotting was performed using RIPA buffer (10 mM Tris-HCl, pH 7.5, 1% NP-40, 0.1% sodium deoxycholate, 0.1% SDS, 150 mM NaCl, and 1 mM EDTA) containing 1% protease and phosphatase inhibitor cocktail, as described previously[^51^](#_ENREF_51). The protein concentrations were assessed by a Bio-Rad reagent (Bio-Rad, PA, USA). The proteins were denatured and run on an SDS-PAGE gel, and immunoblotting was performed as described previously[^52^](#_ENREF_52). The proteins separated by SDS-PAGE were transferred to a nitrocellulose membrane (Schleicher and Schuell Bioscience, Dassel, Germany). Nonspecific binding was blocked by using 5% non-fat dry milk in TBS-T buffer for 1.5 h at room temperature. The membranes were incubated with primary antibody (diluted 1:1,000) overnight at 4°C, washed with TBS-T buffer and further incubated with secondary antibodies (diluted 1:5,000) for 1 h at room temperature. The immunoblot images were obtained by a ChemiDoc^TM^ XRS+ imaging system (Bio-Rad, USA), and protein contents were quantified with Gel-Pro Analyzer software.

**Fatty acid oxidation and synthesis**

HepG2 cells were seeded at a density of 10^6^ cells/well in 6-well plates for 24 h and then cells were lipid-loaded with free fatty acids (400 μM of palmitic acid and 400 μM of oleic acid) with 0.5% bovine serum albumin (GenDEPOT, USA) for 24 h. Lipid-loaded cells were then treated with C3G (10 and 50 μM) or GW7647 (1 μM) as the positive control for another 24 h.

The fatty acid oxidation in HepG2 cells were assessed using [1 − 14C] palmitate. Briefly, HepG2 cells were seeded at a density of 10^4^/well and in 24-well plates for 24 h, then lipid-loaded with free fatty acids (400 μM of palmitic acid and 400 μM of oleic acid) with 0.5% bovine serum albumin (GenDEPOT, USA) for 24 h. The cells were then stimulated with C3G for 24 h, and then incubated with 1.75 mM [1 − ^14^C] palmitate (57 mCi/mM; Perkin Elmer) at 37 °C for 1 h. The ^14^CO_2_ was qualified and the protein concentrations of the resulting lysates were determined for normalization.

Mouse liver FAO was quantified using ^14^C-palmitate. Briefly, fatty acid oxidation in mouse liver was assessed by homogenizing livers in 9 volume of cold 0.25 M sucrose, and centrifuged to gather the supernatant. The liver samples were added with 10% Triton X-100 and then 5 µL of sample was mixed with: 950 µL of 50 mM Tris-HCl (pH 8.0), 10 µL of 20 mM NAD, 3 µL of 0.33 M Dithothreitol, 5 µL of 1.5% BSA, 5 µL 2% Triton X-100, 10 µL of 10 mM CoA, and 10 µL of 1 mM FAD. Reaction was initiated by adding 2 µL of 5 mM palmitoyl-CoA at 37°C, and the signal was determined by a spectrophotometer at wavelength of 340 nm for 5 min. The rate of palmitoyl-CoA oxidation was directly related to the rate of fatty acid oxidation.

For the fatty acid synthesis in HepG2 cells, after removing the medium, cell monolayers were incubated with DMEM containing 1.75 mM [^14^C]acetate (57 mCi/mM; PerkinElmer) at 37°C for 1 h. The medium was removed, and the cells were washed 3 times with ice-cold 0.14 M KCl. Then, 0.75 mL of 1 M NaOH was added, and the cells were collected in test tubes. Ethanol (2 mL) and DW (1 mL) were added to the cells, and the samples were incubated at 90°C for 90 min. Next, 0.5 mL of 7 M HCl and 2.5 mL of petroleum ether were added 3 times to extract lipids, and the samples were dried at room temperature. The radioactivity of the ^14^C-labelled quantity was quantified, and the protein concentrations were determined for normalization.

Fatty acid synthesis in the mouse liver was analysed using fresh livers that were treated with DMEM containing C3G (10 and 50 μM) and 1.75 mM [^14^C]acetate (57 mCi/mM; PerkinElmer) at 37°C for 90 min. Then, fatty acid synthesis in the liver was quantified as described for HepG2 cells.

**Coactivator recruitment assay**

The potential capacity of C3G to activate PPARα, -β/δ and -γ was determined by LanthaScreen^TM^ Time-Resolved Fluorescence Resonance Energy Transfer (TR-FRET) Co-activator Assays (Invitrogen, Carlsbad, CA, USA) as described previously. Serial concentrations of C3G or positive controls (FF, GW0742, and troglitazone) were diluted in the reaction solution according to the manufacturer’s instructions with 1% DMSO and mixed with the LBD of the PPARα, -β/δ and -γ proteins and their coactivator peptides (Fluorescein-PGC1α for PPARα, Fluorescein-C33 for PPARδ/β, and Fluorescein-TRAP220/DRIP-2 for PPARγ). After incubation for 2 h at room temperature, the samples were analysed using a Spectra Max instrument with time-resolved fluorescence laser excitation at 340 nm and emission at 495 nm and 520 nm. Then, the ratio of the emission signals at 520 and 495 nm was plotted against the log of the ligand concentration to generate binding curves. The data were fitted to a sigmoidal dose response curve to determine the half-maximal effective concentration (EC_50_) as provided by GraphPad Prism 8.0 (GraphPad Software, Inc., SanDiego, CA, USA).

**Surface plasmon resonance (SPR)**

The binding affinities of the immobilized PPARα, -β/δ and -γ ligand-binding domains (LBDs) for ligands were evaluated using a Biacore 2000 instrument (GE Healthcare, Uppsala, Sweden) as described previously. All Biacore data were obtained at 25°C using PBS, with 1% DMSO as the running buffer at a constant flow rate of 25 μL/min. After immobilizing the LBDs of PPAR proteins on the CM5 sensor chip (GE Healthcare, Uppsala, Sweden), serial concentrations of samples were automatically injected into flow cells to induce an SPR signal (expressed in response units, RU) as an association rate ('on rate', ka), and then injection of running solution without sample was identified as the dissociation rate ('off rate', kd). The BIA evaluation software version 3.1 (GE Healthcare, Uppsala, Sweden) and a 1:1 Langmuir binding fitting model were used to analyse the equilibrium dissociation constants (K_D_ values).

**Indirect calorimetry**

Mice were orally administered 100 mg/kg body weight/day C3G, and the same volume of distilled water was given to the control group. Oxygen consumption (VO_2_) and carbon dioxide production (VCO_2_) were measured using the Oxylet Physiocage System (Panlab/Harvard apparatus, Cornella, Spain) and the software suite Metabolism (V2.2.01, Panlab). The respiratory exchange ratio used for estimating the respiratory quotient (RQ) was calculated as VCO_2_/VO_2_, and energy expenditure was calculated according to the following formula:

[kcal/(day kg0.75)]=VO_2_·1.44·[3.815+(1.232·RER)].

**Metabolomic analysis**

The plasma and liver samples of mice (HFD and C3G) were obtained at 0, 4, and 8 weeks. For separating the plasma sample from mouse blood, centrifugation at 4℃ and 13,000 rpm for 15 min was used. After that, all samples were kept in a deep freezer at below −70℃ until further analysis. Three types of internal standard compounds, including heptadecanoic acid, were purchased from Sigma Aldrich (St. Louis, MO, USA). N,O-Bis (trimethylsilyl) trifluoroacetamide (BSTFA) with 1% trimethylchlorosilane (TMCS) was purchased from Supelco (Belefonte, PA, USA). 3-*O*-β-d-Glucoside was acquired from Tokiwa Phytochemical (Chiba, Japan). All solvents, such as water, acetone, hexane, and methanol, were of analytical grade (J.T. Baker, Phillipsburg, NJ, USA).

**Metabolic profiling using capillary electrophoresis mass spectrometry (CE-MS)**

***Plasma sample preparation for CE-MS.*** Plasma samples (50 μL) were added to tubes containing 450 μL of methanol with an internal standard (10 μM) and mixed. Chloroform (500 μL) and Milli-Q water (200 μL) were added to the homogenates, mixed thoroughly and centrifuged (2,300 × g, 4℃, 5 min). After centrifugation, the water layer (400 μL) was harvested and filtered with a 5-kDa cut-off filter (Millipore, Ultrafree, MCPLHCC). The filtrates were desiccated and then dissolved in 25 μL of Milli-Q water.

***Liver sample preparation for CE-MS.*** Liver samples were mixed with 1,500 μL of 50% acetonitrile in water containing internal standards (2 μM for cation measurement and 2 μM for anion measurement), homogenized by a multi-sample homogenizer (Shake Master Neo, Bio Medical Science, Tokyo, Japan) and centrifuged. The supernatant was then filtered through 5-kDa cut-off filters (ULTRAFREE-MC-PLHCC, Human Metabolome Technologies, Yamagata, Japan) to remove macromolecules. The filtrate was concentrated by centrifugation and resuspended in 50 μL of ultrapure water immediately before metabolome analysis using CE-MS.

***CE-MS analysis for plasma and liver.*** Cationic component analysis was performed by using an Agilent CE-TOF-MS system (Agilent Technologies, Palo Alto, CA, USA) Machine No. 6. Each sample (2 μL) was injected at 50 mbar for 30 s into a fused silica capillary (50 μm i.d. × 80 cm total length) preconditioned with running buffer (H3301-1001, Human Metabolome Technologies Inc. (HMT), Tsuruoka, Japan), and sheath liquid (H3301-1020, HMT) was delivered. The capillary voltage and MS capillary voltage were set at 27 kV and 4000 V, respectively. Metabolites were scanned in the range of 50-1000 for full-scan mode. Anionic compounds were measured in the positive or negative mode of metabolome analysis using CE-MS/MS on an Agilent CE equipped with an Agilent 6460 TripleQuad LC-MS (Agilent Technologies, Palo Alto, CA, USA) Machine no. QqQ01. Anionic metabolites were also separated through a fused silica capillary (50 μm i.d. × 80 cm total length) preconditioned with a running buffer solution (I3302-1023, HMT, Tsuruoka, Japan), and sample injection was conducted at a pressure of 50 mbar for 25 s. The CE voltage applied was 30 kV. Sheath liquid comprising 50% methanol/water (v/v) was delivered. The analysis was repeated 3 times.

***Identification and quantification of metabolites using CE-MS.*** Cationic metabolites detected in CE-TOF-MS analysis were extracted using the automatic integration software Master Hands (Version 2.13.0.8.h, Keio University), and anionic components in CE-MS/MS analysis were extracted using MassHunter (B.05.00, Agilent Technologies, Santa Clara, CA, USA) to obtain peak information, including m/z, migration time (MT), and peak area. The peak area was then converted to the relative peak area.

**Fatty acid profiling using gas chromatography-time-of-flight mass spectrometry (GC-TOF-MS)**

***Plasma sample preparation for GC-TOF-MS.*** The method of Wang et al. (2009) was slightly modified in the present study. For analysis, plasma samples in the same group were all pooled. Plasma (30 μL) was extracted with 500 μL of cold methanol, and 4 internal standard compounds (30 μL), such as heptadecanoic acid [50 ppm (w/v) in hexane], for lipids were added. Before centrifugation for 15 min at 3,000 rpm and 4℃, the mixture was agitated using a Vortex-Genie 2 (Scientific Industries Inc., Bohemia, NY, USA) for 1 min and stored on ice for 10 min. An aliquot (400 μL) of supernatant liquid was evaporated to dryness in a Centri-Vap (Labconco Co., Kansas City, MO, USA) for 8 h. Then, the dry extracts were derivatized by 30 μL of methoxyamine-HCl [20 mg/mL in pyridine] at 30℃ for 60 min in a Centri-Vap to obtain methyl oxime derivatives, followed by 50 μL of N,O-bis (trimethylsilyl) trifluoroacetamide (BSTFA) with 1% trimethylchlorosilane (TMCS) at 70℃ for 60 min in a Centri-Vap.

***Liver sample preparation for GC-TOF-MS****.* Pooled liver tissues were homogenized in 1 M sodium chloride aqueous solution. A total of 150 μL of liver homogenate was used to analyse metabolites, and the rest of the process was the same as that described for the plasma samples.

***GC-TOF-MS analysis of plasma and liver samples.*** Derivatized metabolites were analysed using an Agilent 6890N GC (Agilent, Palo Alto, CA, USA) coupled to a Pegasus Ⅲ time-of-flight (TOF) mass spectrometer (MS) (Leco, St. Joseph, MI, USA) with an autosampler. Metabolites were separated through a DB-5MS column (30 m length × 0.25 mm i.d. × 0.25 μm film thickness, J and W Scientific, Folsom, CA, USA) in splitless mode with helium as the carrier gas at a flow rate of 1.0 mL/min. Two microliters of the derivatized sample was injected. The oven temperature was started at 85℃, held for 5 min and raised to 205℃ at a rate of 8℃/min. After maintaining the oven for 5 min at 205℃, the temperature was elevated at a rate of 8℃/min to 320℃, which was maintained for another 5 min. The injector and detector transfer line temperatures were 230℃ and 250℃, respectively. Full mass scan ranges were 45-550 *m/z,* and electron impact mass spectra were obtained at 70 eV. The analysis was repeated 3 times.

***Identification and quantification of metabolites using GC-TOF-MS****.* Each metabolite was positively identified by matching its retention time and mass spectrum with those of the authentic standard compounds or with online libraries such as the Wiley 7n mass spectral database (Hewlett-Packard, Palo Alto, CA, USA, 1995), NIST05 MS Library and MS search Program V.2.0s (NIST, 2005) when authentic chemicals were not available. The levels of metabolites in the specimens were calculated relative to those of the internal standard.
