# Supplementary material for: A dietary anthocyanin cyanidin-3-O-glucoside binds to PPARs to regulate glucose metabolism and insulin sensitivity in mice
Source: Commun Biol. 2020 Sep 18;3:514. doi: 10.1038/s42003-020-01231-6 (PMC7501857; doi:10.1038/s42003-020-01231-6)
Supplement: Supplementary file 2 — Description of Additional Supplementary Files [file 42003_2020_1231_MOESM2_ESM.pdf]

Description of Additional Supplementary Files

File Name: Supplementary Data 1
Description: Source data for Figures 1 to 5 and Table 1
