# Supplementary material for: A dietary anthocyanin cyanidin-3-O-glucoside binds to PPARs to regulate glucose metabolism and insulin sensitivity in mice
Source: Commun Biol. 2020 Sep 18;3:514. doi: 10.1038/s42003-020-01231-6 (PMC7501857; doi:10.1038/s42003-020-01231-6)
Supplement: Supplementary file 4 — Reporting Summary [file 42003_2020_1231_MOESM4_ESM.pdf]

nature research

Corresponding author(s): Sung-Joon Lee

Last updated by author(s): Jun 9, 2020

Reporting Summary

Nature Research wishes to improve the reproducibility of the work that we publish. This form provides structure for consistency and transparency in reporting. For further information on Nature Research policies, see Authors & Referees and the Editorial Policy Checklist .

Statistics

For all statistical analyses, confirm that the following items are present in the figure legend, table legend, main text, or Methods section.

n/a	Confirmed	
☐	☒	The exact sample size (*n*) for each experimental group/condition, given as a discrete number and unit of measurement
☐	☒	A statement on whether measurements were taken from distinct samples or whether the same sample was measured repeatedly
☐	☒	The statistical test(s) used AND whether they are one- or two-sided<br>*Only common tests should be described solely by name; describe more complex techniques in the Methods section.*
☐	☒	A description of all covariates tested
☐	☒	A description of any assumptions or corrections, such as tests of normality and adjustment for multiple comparisons
☐	☒	A full description of the statistical parameters including central tendency (e.g. means) or other basic estimates (e.g. regression coefficient) AND variation (e.g. standard deviation) or associated estimates of uncertainty (e.g. confidence intervals)
☐	☒	For null hypothesis testing, the test statistic (e.g. *F*, *t*, *r*) with confidence intervals, effect sizes, degrees of freedom and *P* value noted<br>*Give P values as exact values whenever suitable.*
☒	☐	For Bayesian analysis, information on the choice of priors and Markov chain Monte Carlo settings
☒	☐	For hierarchical and complex designs, identification of the appropriate level for tests and full reporting of outcomes
☒	☐	Estimates of effect sizes (e.g. Cohen's *d*, Pearson's *r*), indicating how they were calculated

Our web collection on statistics for biologists contains articles on many of the points above.

Software and code

Policy information about availability of computer code

Data collection	No software was used.
Data analysis	GraphPad Prism 8.0 (GraphPad Software, Inc., San Diego, Ca, USA).<br>ANOVA was performed using SPSS (Version 12.0, Chicago, IL, USA).<br>Multivariate statistical analyses, such as principal component analysis (PCA) and principal component analysis (PLSR), were performed with SIMCA-P+ (Version 11.0 Umetrics, Umeå, Sweden).

For manuscripts utilizing custom algorithms or software that are central to the research but not yet described in published literature, software must be made available to editors/reviewers. We strongly encourage code deposition in a community repository (e.g. GitHub). See the Nature Research guidelines for submitting code & software for further information.

Data

Policy information about availability of data

All manuscripts must include a data availability statement. This statement should provide the following information, where applicable:
- Accession codes, unique identifiers, or web links for publicly available datasets
- A list of figures that have associated raw data
- A description of any restrictions on data availability

We have provided a full data availability statement in the manuscript.

Field-specific reporting

Please select the one below that is the best fit for your research. If you are not sure, read the appropriate sections before making your selection.

Life sciences study design

All studies must disclose on these points even when the disclosure is negative.

Sample size	This study selected sample sizes based on previous studies. In addition, the accuracy of the research hypothesis and results was verified by repeated experiments.
Data exclusions	Data were not excluded from this study.
Replication	All experiments were replicated independently to verify the reproducibility of the results, and all relevant attempts were successful.
Randomization	Mice were randomly assigned to groups.
Blinding	In this experiment, human subjects are not involved and blinding was not possible.

Reporting for specific materials, systems and methods

We require information from authors about some types of materials, experimental systems and methods used in many studies. Here, indicate whether each material, system or method listed is relevant to your study. If you are not sure if a list item applies to your research, read the appropriate section before selecting a response.

Materials & experimental systems

n/a	Involved in the study	
☐	☒	Antibodies
☐	☒	Eukaryotic cell lines
☒	☐	Palaeontology
☐	☒	Animals and other organisms
☒	☐	Human research participants
☒	☐	Clinical data

Methods

n/a	Involved in the study	
☒	☐	ChIP-seq
☒	☐	Flow cytometry
☒	☐	MRI-based neuroimaging

Antibodies

Antibodies used	PDK4 (Santa Cruz, CA, USA, sc-14495); PDH1a (Abcam, Cambridge, UK, ab10330); p-PDH1a (Abcam, Cambridge, UK, ab92696); b-Actin (Santa Cruz, CA, USA, sc-47778)
Validation	PDK4 (Santa Cruz, CA, USA, sc-14495) is reacted with mouse, rat, and human origin by Western Blotting (goat polyclonal antibody, 47kDa).<br>PDH1a (Abcam, Cambridge, UK, ab10330) is reacted with mouse, rat, and human origin by Western Blotting (mouse monoclonal antibody, 43kDa).<br>p-PDH1a (Abcam, Cambridge, UK, ab92696) is reacted with mouse and human origin by Western Blotting (rabbit polyclonal antibody, 43kDa).<br>b-Actin (Santa Cruz, CA, USA, sc-47778) is reacted with mouse, rat, and human origin by Western Blotting (mouse monoclonal antibody, 43kDa).

Eukaryotic cell lines

Policy information about cell lines

Cell line source(s)	HepG2, Human hepatocellular carcinoma<br>C2C12, Mouse myoblast
Authentication	HepG2 (Human hepatocellular carcinoma, Korean Cell Line Bank, KCLB NO. 88065)<br>Cell line STR Profile (D3S1358: 15,16 vWA: 17 FGA: 22,25 Amelogenin: X,Y TH01: 9 TPOX: 8,9 CSF1P0: 10,11 D5S818: 11,12 D13S317: 9,13 D7S820: 10)<br>C2C12 (Mouse myoblast, ATCC, CRL-1772)
Mycoplasma contamination	Cells were not contaminated with mycoplasma.
Commonly misidentified lines (See ICLAC register)	As a result of ICLAC verification, no cell lines were misidentified in this study.

Animals and other organisms

Policy information about studies involving animals; ARRIVE guidelines recommended for reporting animal research

Laboratory animals	C57BL/6J male mice were purchased from Samtako Co. (Kyunggido, Korea), and PPARα-deficient male mice were purchased from Taconic (Hudson, NY, USA).
Wild animals	This study did not involve wild animals.
Field-collected samples	All mice were maintained under a 12-h light/12-h dark cycle at a temperature of 21-25°C and a relative humidity of 50-60%. At the end of the experimental period, the mice were fasted for 12 h and sacrificed at the same atmosphere.
Ethics oversight	All animal experiments were performed according to a protocol approved by the Animal Experiment Committee of Korea University (Protocol No. KUIACUC-20090420-4).

Note that full information on the approval of the study protocol must also be provided in the manuscript.
